# Sensorimotor processing for balance in spinocerebellar ataxia type 6

**DOI:** 10.1002/mds.26227

**Published:** 2015-04-16

**Authors:** Lisa M. Bunn, Jonathan F. Marsden, Daniel C. Voyce, Paola Giunti, Brian L. Day

**Affiliations:** ^1^ Sobell Department of Motor Neuroscience and Movement DisordersUCL Institute of NeurologyLondonUK; ^2^ School of Health ProfessionsPeninsula Allied Health CentreUniversity of PlymouthUK; ^3^ Department of Molecular NeuroscienceUCL Institute of NeurologyLondonUK

**Keywords:** Motor control, balance, cerebellum, spinocerebellar ataxia, clinical neurophysiology

## Abstract

**Background:**

We investigated whether balance impairments caused by cerebellar disease are associated with specific sensorimotor processing deficits that generalize across all sensory modalities. Experiments focused on the putative cerebellar functions of scaling and coordinate transformation of balance responses evoked by stimulation of single sensory channels.

**Methods:**

Vestibular, visual, and proprioceptive sensory channels were stimulated in isolation using galvanic vestibular stimulation, moving visual scenery, and muscle vibration, respectively, in 16 subjects with spinocerebellar ataxia type 6 (SCA6) and 16 matched healthy controls. Two polarities of each stimulus type evoked postural responses of similar form in the forward and backward directions. Disease severity was assessed using the Scale for Assessment and Rating of Ataxia.

**Results:**

Impaired balance of SCA6 subjects during unperturbed stance was reflected in faster than normal body sway (*P* = 0.009), which correlated with disease severity (*r* = 0.705, *P* < 0.001). Sensory perturbations revealed a sensorimotor processing abnormality that was specific to response scaling for the visual channel. This manifested as visually evoked postural responses that were approximately three times larger than normal (backward, *P* < 0.001; forward *P* = 0.005) and correlated with disease severity (*r* = 0.543, *P* = 0.03). Response direction and habituation properties were no different from controls for all three sensory modalities.

**Conclusion:**

Cerebellar degeneration disturbs the scaling of postural responses evoked by visual motion, possibly through disinhibition of extracerebellar visuomotor centers. The excessively high gain of the visuomotor channel without compensatory decreases in gains of other sensorimotor channels provides a potential mechanism for instability of the balance control system in cerebellar disease. © 2015 The Authors. Movement Disorders published by Wiley Periodicals, Inc. on behalf of International Parkinson and Movement Disorder Society.

Balance disorders are commonly observed after cerebellar lesions arising from genetic causes, ischemia, tumors, alcoholism, and trauma.^1^, ^2^, ^3^, ^4^ However, we have no clear understanding of the cerebellum's role in balance control or the range of fundamental deficits that might be caused by different cerebellar lesions. Balance control involves acting on information about the body's current state of stability signaled by multiple sensory modalities. The cerebellum has the potential to participate in this process, because it either directly or indirectly receives considerable multisensory information known to be important for balance, including that from vestibular,^5^ proprioceptive,^6^ somatosensory,^7^ and visual^8^ sources. Here we pursue this idea by asking whether cerebellar disease is accompanied by a specific deficiency of sensorimotor processing for balance, and if so, whether the deficiency generalizes across all sensory modalities. To examine these questions, we studied a cohort of patients with spinocerebellar ataxia type 6 (SCA6). SCA6 causes death of Purkinje cells in the superior and anterior parts of the cerebellum and gliosis in the flocculo‐nodular lobe.^9^, ^10^ but with little or no extracerebellar involvement.^10^ Thus, it is a rare but well‐defined and relatively pure form of cerebellar degeneration, which during quiet stance causes clear balance impairments that scale with disease severity.^4^

The classical approach for studying balance is to perturb the body and measure the ensuing response. However, natural perturbations of the body inevitably stimulate multiple sensory systems simultaneously, making it difficult to analyze the processing of information from each sensory channel. The approach we have adopted, therefore, is to stimulate each of the three main sensory channels (visual, vestibular, and proprioceptive) in isolation, using stimuli that do not directly perturb the body but that nonetheless produce well‐defined postural responses. The three modes of stimulation were chosen to produce similar postural responses in the same directions so that any differences in response behavior could be attributed to the sensory channel rather than to the motor system generating the response.

We consider two fundamental sensorimotor functions that have been proposed for the cerebellum, namely, control of response scaling^11^, ^12^, ^13^ and coordinate transformation.^14^, ^15^, ^16^ If response scaling of a sensorimotor loop is deficient, the amplitude of the balance response will be either too small or too large. With a deficiency in the coordinate transformation of information from a sensory to an action coordinate frame, such as from head coordinates to leg coordinates, the direction of the balance response may be incorrect or excessively variable.

## Methods

Procedures were approved by the University College London Hospitals NHS Trust ethics committee, and consent was obtained from participants in accordance with the declaration of Helsinki (2004).

### Subjects

Sixteen subjects with SCA6 from different families were recruited from the Ataxia Centre at the National Hospital of Neurology and Neurosurgery. Sixteen healthy control subjects (HC) were recruited from a local advertisement and acted as controls matched to patients by age, height, and weight.

Subjects with SCA6 were included if they 1) were 18 y of age or older, 2) had a confirmed genetic diagnosis of SCA6; 3) had a score greater than zero on the Scale for Assessment and Rating of Ataxia^17^ (SARA) or nystagmus. Note that subject 3 scored zero on the SARA but had nystagmus and a subjective feeling of unsteadiness. Subjects in either group were excluded if they 1) were unable to walk 10 m unaided; 2) were unable to stand independently for 10 s with their eyes closed; 3) were taking drugs (medication or alcohol) with side effects of dizziness, drowsiness, or muscle weakness; or 4) had current or past medical conditions, other than SCA6, that could affect balance. No subjects reported headaches or migraines within the week before testing.

### Clinical Rating of Disease Severity and Sensory Function

The SCA6 subjects were assessed using SARA to provide a measure of disease severity (score: 0 = no ataxia, 40 = most severe ataxia). The Inventory of Non‐Ataxia Symptoms was used to screen for non‐ataxia signs.^18^ Sensory examination was carried out in all SCA6 subjects. Magnetic resonance imaging reports were reviewed to ensure that only those with restricted cerebellar atrophy were included. Ocular examinations were undertaken and the presence of clinically detectable abnormal features recorded, such as nystagmus, oscillopsia, broken smooth pursuit, and ophthalmoplegia. Biosthesiometer ascending and descending threshold measures of vibration sensitivity were collected over the central tibialis anterior (TA) and medial gastrocnemius (mGAS) muscle bellies, and monofilament tests of sensitivity to light pressure (10 g) were collected using the standardized procedures outlined previously.^19^ Measures of near visual acuity and nature of spectacle use was documented (near/distance correction, uni/bifocal). Subjects were asked whether they had ever experienced or were currently experiencing any vertigo symptoms (dizziness, spinning, nausea, migraines).

### Instrumentation

Subjects stood on a force plate (model 9286AA, Kistler, Winterthur, Switzerland) that recorded ground reaction forces. Whole‐body motion was recorded using a three‐dimensional motion‐capture system (CODA, Charnwood Dynamics, Rothley, UK). Rigid clusters of four infrared emitting diodes were fixed using non‐slip elastic straps to the head, the torso (level of C7 vertebrae), and the back of the pelvis, and three diodes were attached to each shank and each foot. All signals were synchronized and sampled at 200 Hz.

### Procedure

Unperturbed body sway was initially recorded for 40 s while subjects stood with a 4‐cm stance width (distance between medial borders of the feet), facing a wall at a distance of 2 m with eyes open. This provided a measure of baseline instability under conditions that were shown previously to give the best correlation with clinical disease severity.^4^ For perturbation trials, the stance width was increased to 8 cm, because patients were more stable and therefore found it less tiring when standing for prolonged periods. However, as shown previously,^4^ this increase had no effect on baseline body sway in the anteroposterior direction.

The three main sensory modalities were investigated using sensory perturbation techniques: 1) visual perturbations in the form of visual motion stimuli (MVS; cw, clockwise; ccw, counter‐clockwise), which leads to a postural response in the same direction as the scene movement^20^; 2) vestibular perturbations using galvanic vestibular stimulation (GVS; r+, anode right cathode left; l+, anode left cathode right)), which evokes a postural response in the direction of the anodal ear^21^; 3) proprioceptive perturbations using muscle vibration (VIB; ts, triceps surae; ta, tibialis anterior) of lower leg muscles, which leads to a postural response in a direction that shortens the vibrated muscle.^22^ To compare across sensory modalities, the postural responses were designed to be similar in form, magnitude, and direction for a healthy standing subject. The response directions that could be studied were constrained by the vibratory stimuli, which were applied to ankle flexors and extensors to produce postural responses in the anteroposterior direction. For vestibular and visual stimuli to evoke responses also in the anteroposterior direction, the head was rotated in yaw through 90 degrees (GVS response is directed approximately along the interaural line), and the visual scene movement was limited to rotation in the sagittal plane about the ankle axis. Technical details of the various stimuli employed are given in Supplemental Data.

For the perturbation trials, subjects stood with their feet 8 cm apart and head rotated to the right through 90 degrees to face the visual scene 0.4 m away in the sagittal plane. This scene remained stationary in all conditions expect for the moving visual stimulus condition. Subjects wore spectacles or contact lenses if required and a visual field restrictor that limited vision to a 74‐degree horizontal viewing angle and 32‐degree vertical viewing angle (approximating a 60 × 25‐cm visible screen area). Earplugs (32 dB) and background white noise masked equipment‐related noise. The subject wore a safety harness that prevented vertical drops of 5 cm or more.

After a random baseline period of 3 to 4 s, a 2‐s sensory stimulus was given followed by a 5‐s post‐stimulus period. Twenty trials of each stimulus (10 per direction) were randomly intermixed with 20 no‐stimulation trials. Trials were randomized according to stimulus type and its direction. Audible tones signaled the start and the end of each trial. Sufficient time was provided between stimuli to allow subjects to adopt the standardized starting position. Rests were included as required during the tests.

### Measurement

Body motion was measured from body displacement approximately at the level of the C7 vertebra. This was converted to an angular measure, using the height of the marker‐cluster above ground level. Stimulus‐evoked response mean magnitude and direction were measured from each subject's mean traces between 0.2 s and 1.0 s (responses to the moving visual scene [MVS] were also measured at 2 s). Direction variability and habituation were measured from single‐trial responses. Baseline sway speed was calculated from the 40‐s period of quiet stance as total horizontal‐plane path/duration as described previously.^7^ See Supplemental Data for measurement details.

### Statistical Analysis

Between‐group comparisons of response magnitudes were carried out using two‐tailed Student's *t* tests for independent samples (PASW Statistics 18, IBM, Armonk, NY, USA). Equal variances were not assumed if Levene's test of equality of variances yielded *P* values less than 0.05. Differences between groups were tested separately for each sensory stimulation mode (GVS, MVS, VIB) and direction (forward, backward), yielding six comparisons for each measure. To account for multiple comparisons, the significance level was set at *P* < 0.01. Associations between response magnitude and disease severity (SARA score) were determined by using Pearson's correlation coefficient.

Analyses of response direction were performed using circular‐data statistical procedures described in Supplemental Data.

## Results

Anthropometric data, clinical assessments, and baseline sway speed are detailed in Table 1. All SCA6 subjects scored zero on the Inventory of Non‐Ataxia Symptoms scale, indicating no clinically detectable non‐ataxia symptoms, and all displayed horizontal gaze‐evoked nystagmus with saccadic pursuit.

**Table 1:** Subject anthropometrics and baseline clinical measures related to disease severity of SCA6 and sensory function

Group	Subject	Sex	Age	Height (m)	Weight (kg)	SARA	SARA Change	Bio_TA	Bio_mGAS	Monofil	Vision	Vertigo	Sway Speed (deg/s)
SCA6	1	M	65	1.78	76.5	9.5	NA	22.7	16.8	9.5	1.8	N ‐ PM	0.25
	2	F	71	1.64	67.8	11	3	20	24.3	10	1.25	N ‐ PMHT	0.28
	3	F	43	1.57	52.9	0	0	12	13.3	10	0.45	N	0.17
	4	F	70	1.65	65.5	17	1.6	23.3	41.3	9.5	1.25	N ‐ PMHT	0.56
	5	F	70	1.65	65.5	17	5.1	23.3	41.3	9.5	1.25	N ‐ PMHT	0.32
	6	F	67	1.6	63.5	14	0.8	12.5	19.5	10	3.2	N	0.66
	7	M	62	1.6	76.6	6	NA	10	25.8	10	3.6	N	0.22
	8	M	40	1.83	63.5	17	1.5	14.3	14.3	10	0.4	N	1.10
	9	F	66	1.61	54.9	13	1.8	17.7	33.8	9.5	2.6	N ‐ PMHT	0.39
	10	F	68	1.6	79.9	13	1.5	10.2	30.9	10	1.5	N	0.30
	11	M	60	1.72	74.5	7	NA	30.8	31	10	6.3	N ‐ PMHT	0.21
	12	M	65	1.83	81.5	9.5	2.1	10.7	20.5	10	1.6	N	0.44
	13	F	73	1.55	78.9	22	2.8	12	33.1	10	3.2	N ‐ PH	1.00
	14	M	62	1.8	86	12	NA	28.7	19.3	10	2.25	N ‐ PH	0.51
	15	M	46	1.69	77.6	20.5	2.2	11.3	14	10	1.125	N	2.21
	16	F	68	1.52	79.4	3	0.7	21	35	9.75	0	N ‐ PMHT	0.42
**SCA6**	**Mean**	**9F : 7M**	**62.3**	**1.67**	**71.5**	**12.0**	**1.9**	**17.5**	**25.9**	**9.9**	**2.0**		**0.56**
	**CI (low, high)**		**57.2, 67.3**	**1.62, 1.71**	**66.7, 76.3**	**9.0, 14.9**	**1.3, 2.6**	**14.2, 20.9**	**21.2, 30.5**	**9.8, 10.0**	**1.2, 2.7**		**0.31, 0.82**
**HC**	**Mean**	**8F : 8M**	**60.3**	**1.69**	**75.3**	**0.0**	**0.0**	**18.5**	**21.6**	**9.8**	**1.7**		**0.23**
	**CI (low, high)**		**55.1, 65.5**	**1.64, 1.75**	**69.4, 81.2**	**NA**	**NA**	**15.1, 21.9**	**16.6, 26.6**	**9.5, 10.0**	**0.8, 2.7**		**0.16, 0.30**

KEY: SARA, Scale for assessment and rating of ataxia (0‐40); SARA change, mean change in SARA per year to date, positive change, worsening disease severity, NA, not available; Bio_TA, mean of bilateral, 3 trial repeats, ascending and descending threshold scores tested over central tibialis anterior muscle bellies; Bio_mGAS, tested over midpoint of gastocnemius heads at the lower border of the Achilles insertion point and over soleus bellies; Monofil, mean monofilament score /10 of bilateral testing; Vision, near visual acuity score held at 40‐cm distance, mean of left and right eyes; Vertigo, current vertigo questioning; N, No current reports of vertigo signs within 1 week; P, Past reports; M, Migraine including dizziness; H, Headaches; T, Travel sickness; Sway speed, trunk sway speed collected over 40 s; CI, 95% confidence intervals.

No group differences in vibration thresholds (TA: *P* = 0.689, mGAS: *P* = 0.225), monofilament testing (*P* = 0.657), or near visual acuity (*P* = 0.704). Mean sway speeds during quiet stance were significantly higher in the SCA6 group (*P* = 0.009). Sway speed correlated with disease severity assessed by SARA (*r* = 0.705, *P* < 0.001).

**Figure 1:**
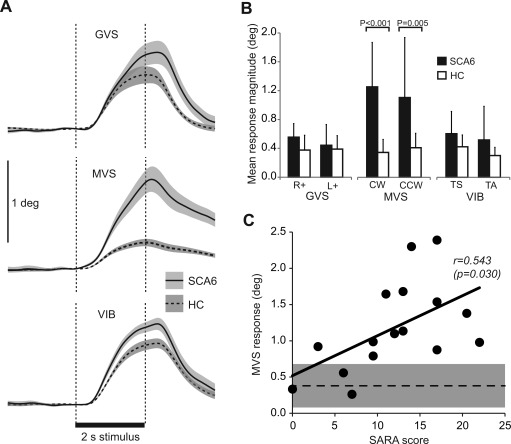
Sensory‐evoked mean response magnitudes. (**A**) Group mean displacement of the body at the level of C7 in the anteroposterior direction to sensory perturbations of vestibular (galvanic vestibular stimulation, GVS), visual (moving visual scene, MVS), and proprioceptive (vibration, VIB) channels. Traces superimposed for cerebellar patients (SCA6, continuous lines) and healthy control subjects (HC, dashed lines). Group mean traces constructed from individual subject mean responses to 10 trials of each polarity of stimulation, combined after inversion of responses to negative polarity stimuli. Shaded areas denote ±1 standard error of the mean. (**B**) Group mean response magnitudes at the level of C7 in the horizontal plane irrespective of direction. Values shown separately for each polarity of the three sensory perturbations comparing cerebellar patients (SCA6, black bars) with healthy control subjects (HC, white bars). Significant difference (*P* < 0.01) between groups present only for MVS. Error bars denote +1 standard deviation. (**C**) Scatter plot of individual SCA6 patients’ mean response to two polarities of MVS (CW and CCW) against their clinical rating of disease severity (SARA). Shaded rectangle shows 95% confidence interval of HC subjects' responses and dashed line denotes the mean. SCA6, spinocerebellar ataxia type 6; CW, clockwise; CCW, counter‐clockwise; SARA, Scale for Assessment and Rating of Ataxia.

Figure 1A shows the time‐course of the sagittal‐plane component of the group mean responses evoked by the three sensory stimuli. The time‐course, magnitude, and direction of responses were deemed sufficiently similar to compare the three sensory modalities.

### Response Magnitude

The group mean response magnitudes are shown for each stimulus modality and polarity in Figure 1B. In general, the SCA6 group tended to show larger responses than controls. The magnitude difference was highly significant for MVS (cw: t[17.55] = 5.67, *P* < 0.001; ccw: t[16.75] = 3.25, *P* = 0.005) but only showed trends for one of the two polarities for VIB (ts: t[22.91] = 2.09, *P* = 0.048; ta: t[16.78] = 1.81, *P* = 0.88) and for GVS (r+: t[29] = 2.51, *P* = 0.018; l+: t[29] = 0.62, *P* = 0.540). The MVS response was measured over a different period compared with the VIB and GVS responses (see Methods). However, when measured over the same time period (0.2‐1 s), the MVS response magnitude combined for the two directions remained highly significantly larger for SCA6 than HC (SCA6, 0.47 ± 0.06; HC, 0.21 ± 0.02; t[20.10] = 4.23, *P* < 0.001).

The magnitude of each single‐trial response was measured to investigate habituation to repeated presentation of the same stimulus. Plots of response magnitude versus stimulus presentation order (shown in Supplemental Data) indicated a uniform lack of habituation. Thus, the response magnitudes to the first and the ninth presentations were not significantly different from each other for all types of stimulus in both groups of subjects (*P* > 0.05 in all cases).

### Response Direction

Statistical analyses of the group mean response directions are shown in Table 2. The response directions were significantly concentrated around a mean direction for all stimulus conditions in both groups. No significant differences were seen in mean response direction for the two groups. The dispersion of response directions around the mean, measured by angular deviation reflecting response direction variability within a group, was not significantly different between groups for any stimulus condition, although a trend was seen for a greater dispersion in SCA6 for the VIB‐ts condition (*P* = 0.023).

**Table 2:** Response directions measured from mean traces of upper trunk displacements

			**GVS**		**MVS**		**VIB**	
			R+	L+	CW	CCW	TS	TA
**SCA6**	N		15	15	16	16	16	16
	Mean direction (°)		−82.95	80.72	−96.30	71.46	−72.59	70.02
	Concentration *r*		0.933	0.944	0.911	0.793	0.877	0.920
	Angular deviation		21.05	19.11	24.15	36.83	28.44	22.92
**HC**	N		16	16	16	16	16	16
	Mean direction (°)		−88.08	72.49	−79.87	71.42	−78.89	82.18
	Concentration *r*		0.937	0.968	0.859	0.764	0.977	0.977
	Angular deviation		20.39	14.44	30.45	39.33	12.22	12.16
**SCA6 vs HC**	Mean direction	*F*	0.43	1.70	2.56	0.00	0.60	3.24
		*P*	0.515	0.203	0.120	0.998	0.443	0.082
	Angular deviation	*U*	141	142	164	147	188	155
		*P*	0.423	0.401	0.184	0.491	0.023	0.323

Notes: One SCA6 subject did not contribute GVS responses because of technical failure. Response direction is reported relative to the visual screen, with 0° indicating motion directly toward the screen, 90° to the left parallel to the plane of the screen, and −90° to the right. All mean directions were highly significantly concentrated (*P* << 0.001). Mean directions compared using Watson‐Williams test. Angular dispersion compared using Wallraff procedure and tested with two‐tailed Mann‐Whitney test. *P* denotes probability, with significance set at *P* < 0.01.

GVS, galvanic vestibular stimulation; MVS, moving visual scene; VIB, muscle vibration; R+, anode right; L+, anode left; CW, clockwise; CCW, counter‐clockwise; TS, triceps surae; TA, tibialis anterior; SCA6, spinocerebellar ataxia type 6; HC, healthy control.

### Within‐Subject Response Direction Variability

Although the directions of the each subject's mean responses were not different for the two groups, possibly the SCA6 subjects were abnormally variable from trial to trial in their response directions. This was quantified by calculating each subject's angular deviation of single‐trial responses. Table 3 gives the group mean and variability of this measure and shows that the within‐subject response direction variability was not significantly different between groups.

**Table 3:** Within‐subject response direction variability (angular deviation, degrees) measured from single trials of upper trunk displacement

		**GVS**		**MVS**		**VIB**	
		R+	L+	CW	CCW	TS	TA
**SCA6**	n	15	15	16	16	16	16
	Mean (°)	31.38	42.31	32.42	45.33	35.02	40.57
	SD	16.84	20.69	15.46	18.49	21.00	19.93
	Median	24.75	49.64	30.69	48.40	36.01	33.13
	Interquartile	28.90	33.40	23.12	17.48	31.43	27.79
**HC**	N	16	16	16	16	16	16
	Mean (°)	26.63	26.48	42.89	42.60	24.92	35.49
	SD	17.34	15.08	16.49	18.24	15.57	16.68
	Median	22.53	22.11	43.13	37.11	22.48	35.95
	Interquartile	16.07	19.60	18.91	22.36	18.42	21.05
**SCA6 vs HC**	*P*	0.446	0.030	0.073	0.616	0.171	0.564

Notes: One SCA6 subject did not contribute GVS responses because of technical failure. Angular deviations compared using two‐tailed Mann‐Whitney test. *P* denotes probability, with significance set at *P* < 0.01.

GVS, galvanic vestibular stimulation; MVS, moving visual scene; VIB, muscle vibration; R+, anode right; L+, anode left; CW, clockwise; CCW, counter‐clockwise; TS, triceps surae; TA, tibialis anterior; SCA6, spinocerebellar ataxia type 6; HC, healthy control.

### Correlation of Response Magnitude With Disease Severity

Response magnitudes were averaged for the two polarities of each stimulus modality and correlated with SARA scores. As shown in Figure 1C, a significant positive correlation was found between SARA and MVS response magnitude (*r* = 0.543, *P* = 0.030), but not for GVS (*r* = 0.108, *P* = 0.702) or VIB (*r* = 0.387, *P* = 0.138).

## Discussion

We have asked whether the balance instability of SCA6 subjects is associated with a deficiency of sensorimotor processing and, if so, whether the deficiency generalizes across all sensory modalities. As reported previously,^4^ balance control of this SCA6 group was abnormal. Even without sensory perturbations, these patients were more unstable than the control group, showing greater body sway during quiet stance, which scaled with disease severity. Despite this instability, many aspects of the responses to single‐channel sensory perturbations were largely unaffected. The notable exception was the response magnitude to the visual perturbation, which was considerably larger than control by a factor of 3 on average. If this excessively large response were attributable simply to the underlying enhanced body sway, then all sensory stimuli should have produced similarly large responses. However, the exaggerated response was reasonably specific to the visual modality and was disease‐related because it correlated with disease severity measured by SARA. This large mean response could not be explained by differences in the rate of habituation of single‐trial responses to repeated presentation of stimuli, because neither group showed significant habituation.

Our results do not replicate those of an earlier study on the visual control of balance in cerebellar patients,^23^ which employed a “moving room” stimulus not dissimilar from our moving visual scene. However, many differences exist between the two studies, including predictability of the stimulus, response direction, measurement period, and, most importantly, the clinical cohorts investigated (SCA6 vs. heterogeneous etiology).

### Specificity and Mechanism of Sensorimotor Disruption

The two processes under consideration were response scaling and coordinate transformation. The positive finding of enlarged balance responses supports the concept of disrupted response scaling. An abnormality of response scaling is not unlike the exaggerated whole‐body response to support‐surface perturbation observed in cerebellar patients^12^ and may represent another expression of typical cerebellar dysmetria reported for limb movements^1^ and eye movements.^24^

A possible explanation for over‐scaling is that it arises from cerebellar disinhibition of sensorimotor centers outside the cerebellum caused by a loss of Purkinje cells, which exert tonic inhibitory influence on deep cerebellar nuclei.^25^ Why the brunt of the abnormality should fall on the visual channel is not clear. It may have something to do with the fact that visual flow is inherently ambiguous in that it signals motion of the environment as well as self and therefore requires a mechanism to extract the self‐motion component for balance control; vestibular and proprioceptive inputs do not require this because they directly signal changes in body state.

An alternative explanation is that the enlarged visuomotor response is a direct result of abnormal cerebellar processing of visual input. The cerebellum receives retinal information indirectly from the accessory optic system and cortically processed visual information via the pons.^26^ Disturbed visual processing has been implicated in other aspects of cerebellar function. Stein^27^ suggested that the cerebellum may play an important role in the visual guidance of movement, whereas some abnormal aspects of limb‐movement trajectories in cerebellar disease have been attributed to aberrant motor responses to visual information.^28^, ^29^ A third possibility is that the over‐scaling results from an indirect visual disruption caused by poor oculomotor control. This could occur if retinal signals are distorted by the abnormal eye movements that were clinically detectable in all of the SCA6 patients studied here.

Some caution is required when interpreting the lack of disruption to coordinate transformation processes. A deficit in spatial transformation of information from a sense‐organ's coordinate frame to an effector's frame would have resulted in detectable direction errors or increased direction variability. However, in this experiment, the spatial relationship between the stimulated sense organs and the body remained fixed throughout testing. A more rigorous test of this process would involve a greater variety of postural changes, for example, by studying a range of head and trunk positions with respect to the feet rather than just the one.

### Can the Visuomotor Disturbance Cause Balance Instability?

One hypothesis for SCA6 balance impairment is that it results from a pure motor disruption, for example, dyssynergia or muscle activation timing problems.^2^, ^19^, ^30^, ^31^ Can an exaggerated visually evoked balance response provide the basis for an alternative sensory hypothesis for balance instability? The neuro‐mechanical system controlling upright stance is often modeled as a mechanical inverted pendulum under sensory feedback control.^32^ One way such a system could go unstable is if the gain of the feedback loops were set too high. The current results could be interpreted as reflecting an excessively high gain of the visual channel without a compensatory decrease in gain of the vestibular and proprioceptive channels, and so is compatible with this hypothesis. However, a simple objection is that cerebellar patients typically become even more unstable when deprived of vision.^23^ Nonetheless, the concept of instability through high feedback gains remains a possibility. This could occur because the relative gains of the different sensory channels are not fixed.^33^ If a sensory channel becomes unavailable, then the gains of the remaining sensory sources may be automatically increased.^34^ The cerebellum has been proposed to play a key role in adaptive gain control, at least for the vestibuloocular reflex,^11^, ^35^ so one could speculate that a loss of the visuomotor loop with eye closure might cause an abnormally large increase in gain of the other sensorimotor loops. More work is required to examine this hypothesis.

### Clinical Implications

The increased gain of the visuomotor feedback loop for balance shown here may be related to the problems encountered by SCA6 patients in daily life. They often report that balance difficulties are particularly severe in busy visual environments, for instance, when walking alongside a busy road or in a crowd. Whatever the cause of the visuomotor disruption in SCA6, it opens an opportunity for targeted rehabilitation of their balance impairment. This could involve training of the oculomotor response^36^ or desensitisation training to respond more appropriately to potentially destabilizing moving visual cues in the environment.^37^

## Author Roles

1. Research Project: A. Conception, B. Organization, C. Execution; 2. Statistical Analysis: A. Design, B. Execution, C. Review and Critique; 3. Manuscript Preparation: A. Writing the First Draft, B. Review and Critique.

L.B.: 1B, 1C, 2B, 3A, 3B

J.M.: 1A, 2C, 3B

D.V.: 1C, 2C, 3B

P.G.: 1A, 1C, 2C, 3B

B.D.: 1A, 1B, 2A, 2B, 3A, 3B

## Financial Disclosures

L.B.: Employment: Plymouth University

J.M.: Employment: Plymouth University

Grants: Progressive MS Alliance; Knowledge Transfer Partnership; VC Community Research Awards; Physiotherapy Research Foundation; Dr William M. Scholl Podiatric R&D Fund; CUC‐ESF; Royal Devon & Exeter NHS Foundation Trust; Plymouth Hospitals

D.V.: Employment: University College London; Organox Ltd.

P.G.: Employment: University College London

Grants: European Community Grant FP7‐HEALTH‐F2‐2010‐242193 (EFACTS)

Honoraria: TAKEDA Cambridge LTD

B.D.: Employment: University College London

Grants: Medical Research Council; Brain Research Trust; UCL Grand Challenge

Honoraria: International Society for Posture and Gait Research

## Supporting information

Additional Supporting Information may be found in the online version of this article at the publisher's web‐site.

Supplementary Information

## References

[1] Stewart G , Holmes G . Symptomatology of cerebellar tumours: a study of forty cases. Brain 1904;27:522‐549.

[2] Holmes G . The symptoms of acute cerebellar injuries due to gunshot injuries. Brain 1917;40:461‐535.

[3] Mauritz KH , Dichgans J , Hufschmidt A . Quantitative analysis of stance in late cortical cerebellar atrophy of the anterior lobe and other forms of cerebellar ataxia. Brain 1979;102:461‐482. 315255 10.1093/brain/102.3.461

[4] Bunn LM , Marsden JF , Giunti P , Day BL . Stance instability in spinocerebellar ataxia type 6. Mov Disord 2012;28:510‐516. 23143967 10.1002/mds.25163

[5] Barmack NH . Central vestibular system: vestibular nuclei and posterior cerebellum. Brain Res Bull 2003;60:511‐541. 12787870 10.1016/s0361-9230(03)00055-8

[6] Matsushita M , Ikeda M . Spinocerebellar projections to the vermis of the posterior lobe and the paramedian lobule in the cat, as studied by retrograde transport of horseradish peroxidase. J Comp Neurol 1980;192:143‐162. 7410609 10.1002/cne.901920110

[7] Joseph J , Shambes G , Gibson J , Welker W . Tactile projections to granule cells in caudal vermis of the rat's cerebellum. Brain Behav Evol 1978;15:141‐149. 638729 10.1159/000123776

[8] Glickstein M . How are visual areas of the brain connected to motor areas for the sensory guidance of movement? Trends Neurosci 2000;23:613‐617. 11137151 10.1016/s0166-2236(00)01681-7

[9] Gomez CM , Thompson RM , Gammack JT , Perlman SL , Dobyns WB , Truwit CL , et al. Spinocerebellar ataxia type 6: gaze‐evoked and vertical nystagmus, Purkinje cell degeneration, and variable age of onset. Ann Neurol 1997;42:933‐950. 9403487 10.1002/ana.410420616

[10] Stevanin G , Dürr A , David G , Didierjean O , Cancel G , Rivaud S , et al. Clinical and molecular features of spinocerebellar ataxia type 6. Neurology 1997;49:1243‐1246. 9371901 10.1212/wnl.49.5.1243

[11] Robinson DA . Adaptive gain control of vestibuloocular reflex by the cerebellum. J Neurophysiol 1976;39:954‐969. 1086347 10.1152/jn.1976.39.5.954

[12] Horak FB , Diener HC . Cerebellar control of postural scaling and central set in stance. J Neurophysiol 1994;72:479‐493. 7983513 10.1152/jn.1994.72.2.479

[13] Manzoni D , Andre P , Pompeiano O . Changes in gain and spatiotemporal properties of the vestibulospinal reflex after injection of a GABA‐A agonist in the cerebellar anterior vermis. J Vestib Res 1997;7:7‐20. 9057156

[14] Manzoni D , Pompeiano O , Andre P . Neck influences on the spatial properties of vestibulospinal reflexes in decerebrate cats: role of the cerebellar anterior vermis. J Vestib Res 1998;8:283‐297. 9652479

[15] Kleine JF , Guan Y , Kipiani E , Glonti L , Hoshi M , Büttner U . Trunk position influences vestibular responses of fastigial nucleus neurons in the alert monkey. J Neurophysiol 2004;91:2090‐2100. 15069099 10.1152/jn.00849.2003

[16] Shaikh AG , Meng H , Angelaki DE . Multiple reference frames for motion in the primate cerebellum. J Neurosci 2004;24:4491‐4497. 15140919 10.1523/JNEUROSCI.0109-04.2004 PMC6729386

[17] Schmitz‐Hübsch T , du Montcel ST , Baliko L , et al. Scale for the assessment and rating of ataxia. Neurology 2006;66:1717‐1720. 16769946 10.1212/01.wnl.0000219042.60538.92

[18] Schmitz‐Hübsch T , Coudert M , Bauer P , et al. Spinocerebellar ataxia types 1, 2, 3, and 6: disease severity and nonataxia symptoms. Neurology 2008;71:982‐989. 18685131 10.1212/01.wnl.0000325057.33666.72

[19] Boulton AJM , Armstrong DG , Albert SF , Frykberg RG , Hellman R , Kirkman MS , et al. Comprehensive foot examination and risk assessment: a report of the task force of the foot care interest group of the American Diabetes Association, with endorsement by the American Association of Clinical Endocrinologists. Diabetes Care 2008;31:1679‐1685. 18663232 10.2337/dc08-9021 PMC2494620

[20] Wolsey CJ , Sakellari V , Bronstein AM . Reorientation of visually evoked postural responses by different eye‐in‐orbit and head‐on‐trunk angular positions. Exp Brain Res 1996;111:283‐288. 8891658 10.1007/BF00227305

[21] Fitzpatrick RC , Day BL . Probing the human vestibular system with galvanic stimulation. J Appl Physiol 2004;96:2301‐2316. 15133017 10.1152/japplphysiol.00008.2004

[22] Polónyová A , Hlavacka F . Human postural responses to different frequency vibrations of lower leg muscles. Physiol Res 2001;50:405‐410. 11551147

[23] Bronstein AM , Hood JD , Gresty MA , Panagi C . Visual control of balance in cerebellar and parkinsonian syndromes. Brain 1990;113:767‐779. 2364268 10.1093/brain/113.3.767

[24] Moschner C , Perlman S , Baloh RW . Comparison of oculomotor findings in the progressive ataxia syndromes. Brain 1994;117:15‐25. 8149209 10.1093/brain/117.1.15

[25] Eccles JC , Ito M , Szentágothai J . The Cerebellum as a Neuronal Machine. Heidelberg, Berlin, and New York: Springer‐Verlag, 1967.

[26] Glickstein M , May JG , Mercier BE . Corticopontine projection in the macaque: The distribution of labelled cortical cells after large injections of horseradish peroxidase in the pontine nuclei. J Comp Neurol 1985;235:343‐359. 3998215 10.1002/cne.902350306

[27] Stein JF . Role of the cerebellum in the visual guidance of movement. Nature 1986;323:217‐221. 3762673 10.1038/323217a0

[28] Beppu H , Nagaoka M , Tanaka R . Analysis of cerebellar motor disorders by visually‐guided elbow tracking movement. 2. Contribution of the visual cues on slow ramp pursuit. Brain 1987;110:1‐18. 3801845 10.1093/brain/110.1.1

[29] Day BL , Thompson PD , Harding AE , Marsden CD . Influence of vision on upper limb reaching movements in patients with cerebellar ataxia. Brain 1998;121:357‐372. 9549511 10.1093/brain/121.2.357

[30] Ivry RB , Keele SW . Timing functions of the cerebellum. J Cogn Neurosci 1989;1:136‐152. 23968462 10.1162/jocn.1989.1.2.136

[31] Bastian AJ , Thach WT . Cerebellar outflow lesions: a comparison of movement deficits resulting from lesions at the levels of the cerebellum and thalamus. Ann Neurol 1995;38:881‐892. 8526460 10.1002/ana.410380608

[32] Mergner T , Maurer C , Peterka RJ . A multisensory posture control model of human upright stance. Prog Brain Res 2003;142:189‐201. 12693262 10.1016/S0079-6123(03)42014-1

[33] Oie KS , Kiemel T , Jeka JJ . Multisensory fusion: simultaneous re‐weighting of vision and touch for the control of human posture. Cogn Brain Res 2002;14:164‐176. 10.1016/s0926-6410(02)00071-x 12063140

[34] Day BL , Cole J . Vestibular‐evoked postural responses in the absence of somatosensory information. Brain 2002;125:2081‐2088. 12183353 10.1093/brain/awf212

[35] Ito M . Cerebellar control of the vestibular neurons: physiology and pharmacology. Prog Brain Res 1972;37:377‐390. 4345129 10.1016/S0079-6123(08)63914-X

[36] Crowdy KA , Kaur‐Mann D , Cooper HL , Mansfield AG , Offord JL , Marple‐Horvat DE . Rehearsal by eye movement improves visuomotor performance in cerebellar patients. Exp Brain Res 2002;146:244‐247. 12195526 10.1007/s00221-002-1171-0

[37] Bunn LM , Marsden JF , Giunti P , Day BL . Training balance with opto‐kinetic stimuli in the home: a randomised controlled feasibility study in people with pure cerebellar disease. Clin Rehabil 2015;29:143‐153. 25082955 10.1177/0269215514539336

