# Supplementary material for: Sensorimotor processing for balance in spinocerebellar ataxia type 6
Source: Mov Disord. 2015 Apr 16;30(9):1259–66. doi: 10.1002/mds.26227 (PMC4949507; doi:10.1002/mds.26227)
Supplement: Supplementary file 1 — Supplementary Information [file MDS-30-1259-s001.docx]

**Sensorimotor processing for balance in spinocerebellar ataxia type 6**

## Supplementary Material:

**Methods**

## *Stimuli*

*Moving visual scene (MVS):* The stimulus comprised of a lightweight (A0 sized 5mm width foamboard) display of alternating black and white 2cm stripes. Constructed as an inverted pendulum, the servo-controlled MVS was positioned to the right of the subject in the sagittal plane and the axis aligned to the medial malleoli of the ankle so that the MVS rotated about the subjects’ ankle joints. The MVS moved 16 degrees and electrical and hydraulic dampeners ensured a constant angular velocity of 8 degrees per second. The scene moved either in the clockwise (cw) or counter-clockwise (ccw) direction and 10 trials of each were recorded.

*Galvanic vestibular stimulation (GVS):* Carbon-rubber electrodes (3cm^2^) were applied bilaterally over mastoid processes with electrode gel (Sigma gel, Parker Lab). The skin around the ears was taped to ensure comfort and localised delivery of current. A 1mA square-wave stimulus was applied for 2s using a custom made current generator. The stimulus polarity was either with right anode-left cathode (r+) or left anode-right cathode (l+), and 10 trials of each were recorded.

*Postural muscle vibration (VIB):* Vibrators were applied bilaterally to the muscle bellies of tibialis anterior and lower muscle bellies of medial and lateral gastrocnemius (overlapping soleus). Vibrators consisted of two 2.5 gram eccentric brass masses on a 8cm axis connected to a 12v DC motor which vibrated at 100Hz. Parts were enclosed in a 10cm (2cm diameter) sealed cylindrical plastic tube which was embedded inside a custom-made silicone mould with 8cm bilateral fixation wings designed to contour around the hemi-circumference of the shank. This assembly was fixed to the subject’s skin using double-sided sticky tape (straps were not used in order to minimise transmission of vibration to antagonist muscle). Vibration was applied either to tibialis anterior (ta) muscles bilaterally or triceps surae (ts) muscles bilaterally, and 10 trials of each were recorded.

## *Measurement*

Body motion was measured from marker data at the level of the top of the torso (the mean 3D-position of the 4 IREDs of the torso cluster, which approximated to the position of the C7 vertebrae) after digital filtering with a low-pass second-order zero-phase 20Hz Butterworth filter using custom written routines in Matlab (MathWorks Inc, Natik, MA, USA). Displacement was normalised to height of the torso cluster above the ground. As the normalising height was a constant value and the horizontal displacement was small relative to the height, this converted the horizontal displacement of the trunk into an angular measure in radians, which then was converted into units of degrees.

Group mean stimulus-evoked response magnitudes and directions were calculated from each subject’s mean traces averaged separately for each condition in the anteroposterior (AP) and mediolateral (ML) directions. To investigate response direction variability and habituation similar measurements were made from single trial responses. Magnitude and direction were measured from changes in marker position from time T1 to T2 and defined as:

$$Magnitude=\surd\left( \left( {AP}_{T2}-{AP}_{T1} \right)^{2}+\left( {ML}_{T2}-{ML}_{T1} \right)^{2} \right)$$

$$Direction=\tan^{-1} \frac{{ML}_{T2}-{ML}_{T1}}{{AP}_{T2}-{AP}_{T1}}$$

For all measurements T1 was set at 0.2s and T2 at 1.0s. However, in some subjects the response to MVS was poorly developed at 1s giving rise to unreliable estimates of response direction. Therefore the MVS response magnitude and direction were also measured with T2 set at 2s, which are the values reported unless indicated otherwise.

Response direction of the body is reported relative to the visual screen, with 0 deg indicating motion directly towards the screen, 90 deg to the left parallel to the plane of the screen, and -90 deg to the right parallel to the plane of the screen.

## *Statistical analysis of response direction*

Analyses of response direction were performed using custom-written procedures based on circular data analyses from Zar^1^. Group mean response directions were measured from the direction of each subject’s mean response per condition. Rayleigh’s test for circular uniformity was applied to establish that response directions within a group were not distributed uniformly around the circle. Once this was established the group circular mean direction and group angular deviation (measure of a group’s between-subject direction variability) were calculated. Group mean directions were compared using the Watson-Williams test. Group angular deviations were compared using the Wallraff procedure and tested with a two-tailed Mann-Whitney test.

Within-subject response direction variability was defined as the angular deviation calculated from the directions of a subject’s single-trial responses per condition. The within-subject angular deviation was compared between groups using a two-tailed Mann-Whitney test.

***Reference***

Zar JH. Biostatistical Analysis, 5th edn. New Jersey: Pearson Education; 2010.

**Results**

**Supplementary Figure**

**Habituation profiles of single-trial response magnitudes.** Group mean response magnitudes versus stimulus presentation order calculated from individual subjects’ single-trial displacement of the body at the level of C7 in the horizontal plane irrespective of direction. Values shown separately for each polarity of the 3 sensory perturbations (galvanic vestibular stimulation, GVS; moving visual scene, MVS; vibration, VIB) comparing cerebellar patients (SCA6, black circles) with healthy control subjects (HC, white squares). Note all 6 stimuli were randomly intermixed and there was no evidence of habituation for either group. Error bars denote +/-1 s.e.m. These mean magnitudes measured from single trials were larger than those measured from averaged responses in Fig.1, particularly for the SCA6 group. This is because single-trial responses include a contribution from the underlying spontaneous body sway, which tends to cancel out in averaged responses.
